# Pathophysiology of the systemic inflammatory response after major accidental trauma

**DOI:** 10.1186/1757-7241-17-43

**Published:** 2009-09-15

**Authors:** Anne Craveiro Brøchner, Palle Toft

**Affiliations:** 1 Department of Anaesthesiology and Intensive Care Medicine, Odense University Hospital, Odense, Denmark; 2 Institute of Clinical Research, University of Southern Denmark, Odense, Denmark

## Abstract

**Background:**

The purpose of the present study was to describe the pathophysiology of the systemic inflammatory response after major trauma and the timing of final reconstructive surgery.

**Methods:**

An unsystematic review of the medical literature was performed and articles pertaining to the inflammatory response to trauma were obtained. The literature selected was based on the preference and clinical expertise of authors.

**Discussion:**

The inflammatory response consists of hormonal metabolic and immunological components and the extent correlates with the magnitude of the tissue injury. After trauma and uncomplicated surgery a delicate balance between pro- and anti-inflammatory mediators is observed. Trauma patients are, however, often exposed, not only to the trauma, but to several events in the form of initial surgery and later final reconstructive surgery. In this case immune paralysis associated with increased risk of infection might develop. The inflammatory response is normalized 3 weeks following trauma. It has been proposed that the final reconstructive surgery should be postponed until the inflammatory response is normalized. This statement is however not based on clinical trials.

**Conclusion:**

Postponement of final reconstructive surgery until the inflammatory is normalized should be based on prospective randomized trials.

## 

A local inflammatory response always occurs in relation to trauma. Severe injury or multiple trauma evoke a systemic inflammatory response. This systemic inflammatory response to major injury is caused by hormonal, metabolic and immunological mediators, and is associated with a haemodynamic response. Accidental unanaesthetised trauma is also to a larger extent associated with ischemia, ischemia/reperfusion (I/R) injury, hypovolemia and the immunological reactions secondary to blood transfusion. The systemic inflammatory response is required for tissue repair and has evolved in all mammals to optimise the healing potential of an organism. In uncomplicated trauma patients the systemic inflammatory response is temporary, predictable and well balanced between pro- and anti-inflammatory mediators. If the patient is exposed to severe major trauma an initial exaggerated proinflammatory response may be observed.

In contrast to the scheduled surgical patient, the trauma patient is exposed to several events or hits. The first hit is the trauma and the second the necessary damage-control surgery. In response to these hits the immune system might be exhausted with increased risk of infection and sepsis. The final reconstructive surgery is often postponed to avoid the detrimental triad of hypothermia, acidosis and coagulopathy, but also to avoid another hit to the immune system. The timing of the final surgery is widely discussed.

Knowledge of the normal inflammatory response to trauma makes it possible for the anaesthetist or surgeon to react if an abnormal response is observed. In this review we will describe the normal inflammatory response to major trauma, the impact of I/R and hypovolemia and the timing of surgery.

This review describes the normalisation of the immune system following trauma in relation to the timing of definitive fracture stabilisation.

## Methods

An unsystematic review of the medical literature was performed and articles pertaining to the inflammatory response to trauma were obtained. The literature selected was based on the preference and clinical expertise of authors.

## The hormonal metabolic response

Major accidental trauma is followed by a hormonal metabolic response. This response is characterized by increased secretion of various stress hormones such as adrenalin and cortisol, but also glucagon, growth hormone, aldosterone and anti-diuretic hormone [1,2].

The adrenocortical response to trauma was first described nearly 100 years ago and has been demonstrated in all investigated mammals ranging from rodent to man [3]. Afferent impulses from the site of injury stimulate the secretion of hypothalamic releasing hormones which further stimulate the pituitary gland. Cortisol is secreted by hormonal stimulation of the adrenalin cortex while adrenalin is secreted by the adrenal medulla in response to activation of the sympathetic nervous system. Noradrenalin spills over into the plasma from the sympatric nerve endings. The magnitude and duration of the hormonal response to traumatic stress correlate well with the extent of the trauma [4]. The neuroendocrine stress response interacts with the immunological response to trauma [5]. There is no evidence that hormonal treatment can improve the outcome following major trauma in humans.

It has been shown in animal studies that estrogen and to a lesser extent dehydroepiandrosterone (a precursor of estradiol and testosterone) is protective in trauma [6,7]. In a trauma hemorrhage shock model, administration of estrogen restored the cardiovascular, hepatocellular, and immune function [8]. In most human observational studies, female gender protects against complications and mortality associated with trauma [6]. However, it remains to be demonstrated that the administration of estrogen also is protective following trauma in humans [9].

Major accidental trauma also induces a metabolic response. Following major trauma the metabolic rate is reduced for a period lasting from several hours up to 24 hours. This is followed by a hypermetabolic and catabolic phase [10] characterized by catabolism of bones, muscle and fat and increased gluconeogenesis resulting in hyperglycaemia [11]. Following uncomplicated major trauma this hypermetabolic phase usually lasts less than a week. This hypermetabolic response is associated with increased oxygen demands in the tissues. Elderly patients with co-morbidities such as chronic obstructive pulmonary disease and cardiac disease have reduced physiological reserves, and might not be able to cope with the increased oxygen demands. Thus lack of these physiological reserves is probably more important in explaining the increased mortality in elderly patients following major trauma than their reduced immune response [12].

If the hypermetabolic response lasts more than 1 or 2 weeks, the patient has probably developed severe systemic inflammatory response (SIRS) and underlying infection and sepsis should be suspected.

## The haemodynamic response

The normal haemodynamic response to major trauma was first described by Cuthbertson [10]. Like the immunological and metabolic response, the haemodynamic response to major trauma is biphasic. The initial shock phase of trauma where haemorrhage causes hypovolemia is characterised by pronounced peripheral vasoconstriction, retention of sodium chloride and water, and a translocation of blood from peripheral to central vital organs. More than 70 years ago, the shock phase in un-resuscitated trauma victims was described as lasting one day [10]. Today the duration of the shock phase is limited due to early goal directed administration of intravenous fluids and blood transfusion. Most anaesthetic agents induce vasodilatation and thus counteract the peripheral vasoconstriction which in the initial shock phase is vital.

When the trauma patient is fluid resuscitated, the haemodynamic response becomes characterised by vasodilatation and increased flow not only to vital organs but also to the muscles and injured tissue. During this flow phase, or hypermetabolic phase, the oxygen consumption and CO_2 _production is increased [13]. The increased metabolism reflects the increased activity of cells repairing the injured tissue. The catabolism of muscles and the gluconeogenesis is also associated with the flow phase. To compensate for the increased oxygen consumption the body reacts with tachycardia, increased cardiac output increased respiratory rate and vasodilatation. It is important during this flow phase to maintain a sufficient intravascular volume by administration of intravenous fluids [14]. In the uncomplicated trauma patient, the flow phase symptoms last only a few days. If the tachycardia, increased respiratory rate, associated leukocytotosis, and increased temperature following major trauma do not normalise within 4-5 days, complications should be suspected [15].

In traumatised tissues the microcirculation is jeopardised due to either direct damage to the vessels or thrombosis. The supply of nutrients to the traumatised tissue is dependent on a concentration gradient as created by hyperglycaemia. In addition to the regional vasodilatation capillary leak with local oedema develops. Revascularisation develops in the traumatised tissue within 3-7 days as observed by Hunt et al [16]. Thus within a week, the capillary leak, hyperglycaemia and oedema will normalise following uncomplicated major trauma.

## The immunological response

A local inflammatory response always occurs in relation to tissue trauma. Local mediators in tissue trauma include kinins and arachidonic acid metabolites. In addition, histamine is released from mast cells in the tissue. These local mediators increase capillary permeability, tissue oedema, and stimulate the local infiltration of immune cells. While most of these local mediators have a short half life, the effects exerted by these mediators are longer lasting, rendering measurements of the concentration of these mediators in serum unimportant as concentrations of mediators do not necessarily reflect the important local activity.

Another endogenous trigger of inflammation is high mobility group box 1 protein (HMGB1). HMGB1, which is released from necrotic or injured cells, attracts neutrophils and macrophages to the site of injury, increases vascular leakage, and reduces the perfusion pressure in the micro circulation [17].

One of the genetically best preserved non-specific reactions to injury or infection is the complement cascade. The complement can be activated by three pathways: by antigen-antibody complexes, by bacterial cell wall components, and by the mannan-binding lectin pathway. The split products of the complement activation are able to lyse bacteria directly, opsonise antigens, attract neutrophils and activate platelets. Activation of the complement system in injury is closely connected to the coagulation cascade.

Following trauma, the coagulation system is early and readily activated. It has been demonstrated that thrombin generated in the coagulation cascade activates C5a in the complement system. Activation of the complement system mediates the immune response. In this way the coagulation cascade is connected to the immune system [18].

However, early in trauma it has been difficult to predict the clinical outcome by measuring products of the complement system [19,20].

**Figure 1:**
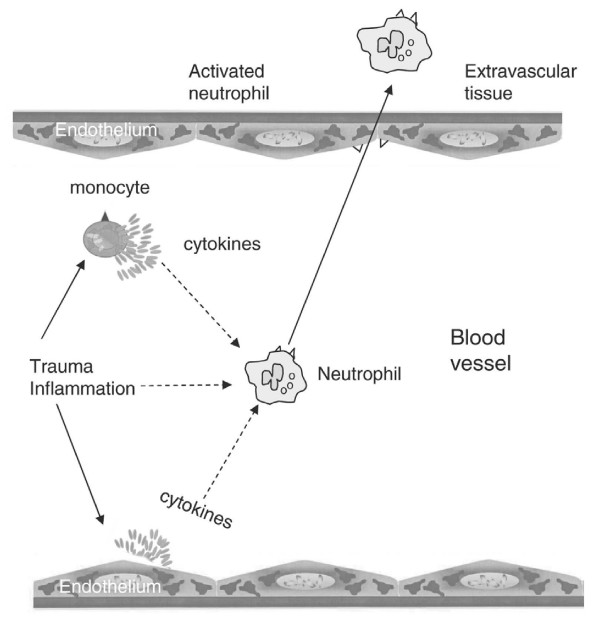
**The proinflammatory response induced by trauma**.

Monocytes and endothelium in the area of injury release proinflammatory cytokines of which the most important are IL-1-β, TNF-α, IL-6, IL-8 and IFN-γ [21] (Fig. 1). The first cytokines secreted after trauma are TNF-α and IL-1. IL-1 and TNF-α are short lived cytokines and have a similar effect on the immune system. The half life of TNF-α and IL-1 is 20 minutes and 6 minutes respectively [22]. TNF-α and IL-1 stimulate many immunological important cells and are able to induce secretion of proinflammatory cytokines such as IL-6 and IL-8, and the anti-inflammatory cytokine IL-10 [23-25]. IL-1 also induces a febrile response. IL-6 is first detectable in plasma within an hour after trauma. IL-6 stimulates the hepatic acute phase protein synthesis with release of C-reactive protein (CRP) and procalcitonin. The secretion of IL-6 correlates with the magnitude of the trauma, the duration of surgery and the risk of postoperative complications [26]. IL-6 has been suggested as a mediator for immune monitoring in the damage control strategy.

IL-6 activates neutrophils and NK-cells and inhibits the apoptosis of neutrophils observed following trauma. Although IL-6 functions as a proinflammatory cytokine in the early hours following trauma, it also has an anti-inflammatory effect by promoting the release of IL-1 receptor antagonists and soluble TNF-receptors [27,28]. IL-6 also induces the production of prostaglandin E2, which stimulates the release of the potent anti-inflammatory cytokine IL-10 [29] (Table 1). In this way the initial proinflammatory response following trauma is soon balanced by a compensatory, anti-inflammatory response syndrome. Also induced by IL-1 and TNF-α is the secretion of IL-8. Initially IL-8 attracts initially neutrophils, but later also monocytes lymphocytes and fibroblasts to the area of injury. IL-8 can activate the neutrophils and it prolongs the half life of neutrophils in the area of injury [30]. If the proinflammatory response becomes exaggerated patients might develop SIRS with increased risk of organ dysfunction. On the other hand if the proinflammatory response is repeated, especially in severely ill patients with significant co-morbidities the immune system may become exhausted with increased risk of infection. The initial high concentration of proinflammatory cytokines and acute phase protein measured within the first hours to a few days following major trauma, will gradually normalize and will be balanced by an anti-inflammatory response. If a second peak of acute phase proteins and proinflammatory cytokines is measured in the circulation, complication such as infection should be suspected. Generally there is a strong association between the extent of the tissue injury and the level of cytokines in plasma, while it has been more difficult to demonstrate a correlation between the cytokine response and mortality in trauma patients [31].

**Table 1:** Mediators of the inflammatory response following trauma

	Secretion stimulated by	Cellular origin	Function	Ref.
IL-1(β)	Activation of macrophages	Released from monocytes and endothelium	Pro-inflammatory Induces fever, secretion of IL-6 and 8	[23]

IL-4	Trauma	Activated T-cells	Anti-inflammatory	[45]

IL-6	IL-1β TNF-α	Released from monocytes and endothelium	Pro- and Anti-inflammatory, production of CRP, procalcitonin. IL1R-antagonist, PGE2	[22,26]

IL-8	IL-1 (β)	Released from monocytes and endothelium	Pro-inflammatory, activate PMN, attracts monocytes, fibroblasts. Prolongs half-life of PMN	[24]

IL-10	PGE2	Released from monocytes and endothelium	Anti-inflammatory	[25]

TNF-α	Activation of macrophages	Monocytes and endothelium	Induces secretion of IL-6 and 8	[22]

IFN-γ	Trauma	NK- cells Activated T-cells	Pro-inflammatory	[42]

HMGB1	Always localized in the nucleus of the cells	Released from nucleus of necrotic cells	Attracts neutrophils and macrophages	[17]

MPO	Activated PMN	Released from granules in monocytes and PMN	Degrades bacteria and cellular debris	[36,37]

Elastase	Activated PMN	Released from granules in monocytes and PMN	Degrades bacteria and cellular debris	[36,37]

Free oxygen radicals	Activated PMN	Released from monocytes and PMN	Degrades bacteria and cellular debris	[36,37]

## The cell mediated immunological response

**Figure 2:**
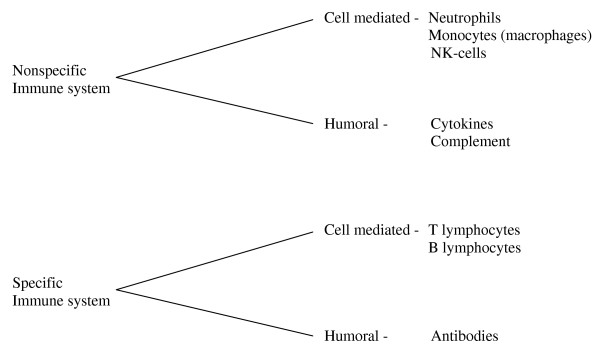
**Brief review of the immune system**.

Major accidental trauma especially affects the non-specific cell mediated immunity. The non-specific cell mediated immunity consists first of all of the neutrophils, but also of monocytes and NK-cells (Fig. 2). The neutrophils are the first cells to arrive at the area of injury [32]. At the same time leucocytosis is observed in peripheral blood [33]. If the accumulation of neutrophils in the tissue becomes exaggerated, immature forms of neutrophils are observed in the peripheral blood. The leucocytosis may be explained partly by the decreased apoptosis observed up to 3 weeks following major trauma [34]. The local migration of neutrophils into the site of tissue damage is important for wound healing and for protection against invading micro-organisms.

Between the resting state and the full activated state of neutrophils, an intermediate state called priming or preactivation exists. In this priming state a second hit can evoke a more exaggerated response [35]. In major trauma, the neutrophils become primed by chemo-attractants in the injured tissue and by exposure to circulating cytokines. In a study by Botha et al. [33], maximum priming of neutrophils was observed 3-24 hours following major trauma. The primed neutrophils are characterized by increased expression of adhesion molecules on their surface. The second hit to the immune system in the trauma patient might be necessary surgery or the development of nosocomial infections.

Following major trauma, the neutrophils do not only accumulate in the injured tissue. A systemic accumulation of neutrophils is also observed. This accumulation of primed neutrophils in non-injured tissue is best understood following infection. Following injection of bacterial products, accumulation of activated neutrophils is observed, especially in the lungs and liver [36]. These neutrophils are protective against infection. The activated neutrophils, however release enzymes such as elastase and myeloperoxidase (MPO) by exocytosis and are capable of oxidative burst activity which may cause damage to uninjured tissue. This systemic accumulation of neutrophils is important in the pathogenesis of tissue damage such as the development of acute respiratory distress syndrome (ARDS) following major trauma. This whole body inflammatory response following major trauma has been described by Nuytinck et al. [37] in an autopsy study. If the stimulation of neutrophils and their accumulation in tissue becomes excessive, impaired function of those neutrophils remaining in the blood stream has been observed. Thus, following excessive activation, some exhaustion may also be observed in the neutrophils.

Increased expression of adhesion molecules is observed on neutrophils as well as on endothelium, which facilitates the trans-migration of neutrophils from the blood stream into the tissue. A migration of neutrophils into the tissue involves several steps: First there is a tethering and rolling along the endothelium, then there is an arrest of the neutrophils, and third a migration into the tissue. Selectin adhesion molecules such as CD62L on the neutrophils are involved in the primary rolling while the integrin adhesion molecules such as CD11b/CD18 mediate the more firm adhesion [38]. The last step of the migration into the tissue is induced by chemo-attractants within the injured tissue. Increased expression of CD11b/CD18 on the surface of neutrophils is measured 24 hours after trauma and increased levels may be observed for the next 1-3 weeks [39].

The second component of the non-specific cell-mediated immunity is the mononuclear phagocyte system. These mononuclear cells are referred to as monocytes when circulating in the bloodstream and macrophages when found in the tissue (i.e.: Kupffer cells in the liver). Monocytes and macrophages are able to phagocytise and generate oxygen free radicals; they have limited secretion of enzymes, but have a very active synthesis of cytokines. In addition they are able to present antigens by the major histocompability complex 2 (MHCII). Earlier studies focused on their phagocytic functions whereas recent reports focus on their synthesis of cytokines and antigen presentation. Several investigators have observed a deactivation of monocytes following major trauma. This deactivation is characterized by a reduced MHCII expression. The reduced expression of MHCII on the surface of monocytes correlates with the extent of trauma, and there is some evidence that it also correlates with later development of sepsis [40]. The reduced expression of MHCII on monocytes has been used to monitor the degree of immune paralysis. After uncomplicated trauma, the expression of MHCII on monocytes is normalized within a week [41]. The reduced MHCII expression can be normalized in traumatized patients by treatment with IFN-γ. IFN-γ did not, however, change incidence of infection or mortality [42]. Also, the ability of the monocytes to produce cytokines is impaired following major trauma. This has been shown in vitro where monocytes from trauma patients were stimulated and afterwards showed a reduced production of cytokines. Thus, following major accidental trauma the monocytes do not show a biphasic pattern. In contrast, the function of monocytes is continuously decreased. This deactivation of monocytes with reduced expression of MHCII and decreased ability to secrete cytokines is measured simultaneously with increased secretion of pro- as well as anti-inflammatory cytokines and activation of neutrophils.

The third component of the non-specific cell mediated immunity is the NK cells. Following a modest trauma, the NK-cell activity is reduced for only several days [31]. Following major trauma such as thermal injury, the NK-cell activity has been observed to be suppressed for 2-4 weeks [43].

T-lymphocytes are part of the specific cell-mediated immunity and are less affected by trauma. The functions of T-lymphocytes can be measured as the delayed type hypersensitivity (DTH) or their ability to proliferate upon stimulation. Subpopulations of T-cells are able to kill any cell which presents an appropriate antigen. DTH as well as T-cell proliferations are suppressed following trauma [44].

In recent years, attention has, however, been focused on the Th1/Th2 lymphocyte ratio. The reduced production of IFN-γ from NK-cells favours a shift of the Th-1/Th-2 lymphocyte ratio towards a Th-2 dominated cytokine pattern. The Th-2 lymphocyte cytokine pattern is characterized by a production of the anti-inflammatory cytokine IL-4 and IL-10. This shift in balance towards Th-2 lymphocyte dominance induces a down regulation of the cell mediated immunity and is part of the counter inflammatory response [45].

## Hemorrhagic shock and the immune system

Hemorrhagic shock is one of the two principal reasons of death after trauma. In trauma death, three peaks are encountered. The first is within the first hour. The second peak is within the next 24 hours where hemorrhagic shock contributes considerably to the high mortality. The last mortality peak occurs after days or weeks and the cause of death is often SIRS or MODS and will not be discussed in the present review.

Not much is known about the human inflammatory response during the first hour after hemorrhagic shock. One important limitation is the difficulty of obtaining blood samples. Thus, in this first hour, mainly animal studies are available.

Immediately after vascular damage and haemorrhage, leucocytes begin rolling along the activated endothelium. In haemorrhage complement is activated. The secretion of HMBG1 and the production of cytokines are initiated [46]. Proinflammatory cytokines are produced following haemorrhage and continued high levels of IL-6 correlates with mortality in hemorrhagic shock. Recent studies indicate that male gender may be associated with higher levels of IL-6 and thus poorer outcome [47]. The anti-inflammatory cytokine IL-10 is also secreted. Recent studies have shown that IL-10 deficiency augments acute lung injury in hemorrhagic shock [48].

In animal studies, it has been shown that blood loss quite often initiates a suppression of NK-cell activity. In one animal study performed by Yago et al. [49], moderate trauma did not depress the NK-cell activity, but impairment of NK-cell activity correlated with the amount of blood loss. Transfusion of blood further induces a suppression of the immune system after major trauma. It has been observed that blood transfusion can induce a shift towards Th-2 lymphocyte and a down regulation of MHCII antigen presentation on monocytes. In animal studies, it has also been demonstrated, that blood transfusion in comparison to Ringer solution decreases the NK-cell activity [50]. In human studies, the amount of blood transfusions may reflect the severity of the injury and post-trauma complications might be caused by the severity of injury as much as blood transfusion. In humans, however, several data-bank analyses demonstrated that blood transfusion is an independent risk factor for post-trauma complications [51].

## I/R in relation to trauma and the inflammatory response

I/R is often associated with trauma and in fact all major trauma are expected to include I/R to a certain extent. It is difficult to differentiate between damage caused by I/R, injury and haemorrhage, and to a certain extent they act simultaneously. Global ischemia following major trauma is mainly present due to severe hypotension following arterial and venous bleeding. A major trigger of inflammation following injury is the reperfusion. After the I/R injury, leucocytes are attracted to the affected area. The production of pro- as well as anti-inflammatory cytokines is increased. HMGB1 is also secreted, but the precise role of HMGB1 in I/R is not known as recent studies have shown that preconditioning with HMGB1 reduces the I/R injury [52]. The reperfusion elicits activation and accumulation of neutrophils not only in the damaged tissue, but also in distant organs. The most susceptible organs are the kidneys, the liver and the lungs. When these organs are examined after an I/R injury, neutrophils and a large concentration of MPO are found. MPO catalyses the formation of oxygen free radicals such as hypochlorite, and chloride ions. Oxygen free radicals have a bactericidal capacity but may also injure the local tissue [53].

In I/R all three pathways of complement are activated and contribute to the tissue damage, inserting holes/perforations in the cellular membranes [54].

According to classical immunology T-lymphocytes were not suspected of playing a role in I/R. Animal studies have however shown that knockout mice lacking T-cells were protected against I/R [55].

## Timing of surgery in major accidental trauma

**Figure 3:**
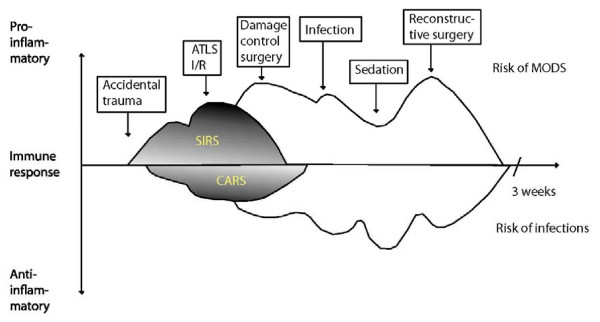
**The pro- and anti inflammatory response to accidental trauma**.

Major trauma induces an inflammatory response initially characterized by increased levels of proinflammatory cytokines and activation of neutrophils. This pathophysiological inflammatory response is determined not only by genetic disposition, physiological states, the type and amount of injury, but also by surgery. In the early hour of major trauma, the patient is resuscitated with advanced trauma life support, treating hypoxia as well as hypovolemia. Resuscitation relieves ischemia in the tissue, but also induces I/R injury. In the early hours following major trauma, life-saving surgical procedures should be performed, for example thoracic drainage, emergency laparotomy, pelvic or abdominal packing and embolization of bleeding vessels. The surgical procedures, the I/R injury, the possible microbiological invasion in the form of aspiration pneumonia or infection of wounds, induce a further activation of the proinflammatory response. Day 1 surgery is limited to damage control interventions, such as stabilisation of long bone fractures, decompression procedures and debridment. In this way, the detrimental triangle of hypothermia, acidosis, and coagulopathy is avoided, and the patient is transferred to the ICU for further stabilisation. But at the same time a further proinflammatory systemic response is also avoided. Normalisation of acidosis, coagulopathy and hypothermia is the basis for the damage control surgery concept [56]. In this context it has been shown that even slight hypothermia increases the perioperative bleeding [57]. While resuscitation, necessary life saving surgical procedures and damage control surgery has to be performed within the first hours or day, the timing of reconstructive surgery, especially orthopaedic surgery, has been widely discussed (Fig. 3).

Several immune modulating trials have been performed to either reduce the initial exaggerated proinflammatory response or stimulate the immune system during later immune paralysis. To reduce the proinflammatory response in trauma patients, recombinant granulocyte colony stimulating factor and indomethacin has been tried [58]. To stimulate the later immune paralysis in trauma patients, treatment with IFN-γ and prostaglandin E2 has been investigated [42]. In clinical practice, however, no immune modulating therapy has been established. To decide whether to stimulate or suppress the immune system, we will need a bedside monitoring assay. Plasma levels of IL-6 have been proposed as a marker of the proinflammatory response, whereas the reduction of the MHCII expression on monocytes has been proposed as a way to monitor the anti-inflammatory response [59,41]. At the present time, we lack a fast-acting biological assay which could measure the state of pro- versus anti-inflammatory balance in the specific trauma patient. Thus, one of the few ways we can modulate the immune system is planning of the reconstructive surgery.

There is general agreement that reconstructive surgery should be postponed until pH, temperature and coagulopathy have normalised. In addition the cardiovascular system should be stable without vasoconstrictors (serum lactate below 2), the oxygenation of the tissue acceptable with low to moderate FiO2, ICP below 20 mm Hg, platelets >100,000 per μl and the diuresis at about 1 ml/kg/hour [60]. It has also been proposed that reconstructive orthopaedic surgery such as definitive osteosynthesis should be postponed until the proinflammatory response has normalized. It has been stated that in general the immune response peaks on day 2 and returns to baseline 6-7 days following major trauma. The immune response is, however, more extended. Investigations following major trauma have demonstrated maximum priming of neutrophils within 3-24 hours [33,39]. Oxidative burst of granulocytes was maximal 6 hours following trauma and returned to baseline 2 weeks later [61]. Most often, reconstructive surgery is performed before this normalisation. Similarly the adhesion molecule CD-11/CD-18 had maximal expression within the first 24 hours following major trauma and was normalized 3 weeks later. The same pattern has been observed with the selectin adhesion molecules. Usually, following uncomplicated major trauma, the expression of MHCII on monocytes is normalized within 1 week [41]. The reduced apoptosis of neutrophils, however, lasts 3 weeks following major trauma [34,61]. If a normalisation of the immune system is required before reconstructive orthopaedic surgery is performed, the reconstructive surgery should be postponed to 3 weeks post-trauma. Such an excessive postponement of reconstructive surgery is not based on randomized controlled trials [60]. Before such a postponement of reconstructive surgery is introduced in clinical practise, a prospective randomised trial should be performed, documenting the beneficial effect. Postponement of surgery is not without risk and non-stable dislocated fractures increase the risk of ARDS and make it difficult to mobilize the patient. Intubated, immobilized patients have increased risk of ventilator associated pneumonia and long-term sedation also has immune suppressive effect. The benefit of postponing reconstructive surgery therefore has to be balanced against these risks [62,63].

## Abbreviations

IL- 1, 4, 6, 8: Interleukin 1, 4, 6,8,10; IFN-γ: Interferron Gamma; TNF-α: Tumor Necrosis Factor alfa; HMGB1: High mobility group box 1 protein; MPO: Myeloperoxidase; PMN: Polymorphonuclear Leucocytes; I/R: Ishemia/reperfusion; SIRS: Systemic Inflammatory Response Syndrome; CRP: C - Reactive Protein; NK-CELL: Natural Killer Cell; ARDS: Acute Respiratory Distress Syndrome; CD 11, 18, 62: Cluster of Differentiation; MHCII: Major Histocompability Complex; MODS: Mullti-organ Dysfunction Syndrome; ICU: Intensive Care Unit.

## Competing interests

The authors declare that they have no competing interests.

## Authors' contributions

ACB and PT both participated in the process by finding articles via Pub Med, writing the manuscript, and designing the figures and tables. Both authors have read and approved the final manuscript.
